# Pleural‐Based *GLI1*‐Altered Mesenchymal Tumor With *ACTB‐GLI1* Fusion: A Rare Pulmonary Entity Mimicking Synovial Sarcoma

**DOI:** 10.1111/1759-7714.70271

**Published:** 2026-03-26

**Authors:** Marco Lizwan, Li Xiao, Chun Yuen Chow, Hiu Yeung Lau, Boon‐Hean Ong

**Affiliations:** ^1^ Department of Cardiothoracic Surgery National Heart Centre Singapore Singapore Singapore; ^2^ Department of Anatomical Pathology Singapore General Hospital Singapore Singapore; ^3^ Department of Pathology and Laboratory Medicine KK Women's and Children's Hospital Singapore Singapore

**Keywords:** *ACTB‐GLI1* fusion, *GLI1*‐altered mesenchymal tumor, *GLI1*‐rearranged tumor, lung, pleura, thoracic surgery

## Abstract

*GLI1*‐altered mesenchymal tumors are exceptionally rare mesenchymal tumors, with recent molecular studies implicating *GLI1* gene fusion or amplification. We report a 39‐year‐old nonsmoking woman who presented with an incidentally detected right pleural‐based mass. Cross‐sectional imaging identified a paraspinal pleural lesion at the T5–T6 level without intraspinal extension. Video‐assisted thoracoscopic resection revealed a biphasic tumor and next‐generation sequencing confirmed an *ACTB‐GLI1* fusion. The diagnosis of *GLI1*‐altered mesenchymal tumor was established. After multidisciplinary discussion, the patient opted for radiologic surveillance instead of adjuvant therapy. She remains disease‐free at 1‐year follow‐up. This case expands the anatomic spectrum of *GLI1*‐altered tumors and underscores the value of molecular testing in distinguishing them from other pleural neoplasms.

## Graphical abstract

Pleural‐based *GLI1*‐altered mesenchymal tumor presenting as an incidental thoracic mass, characterized by biphasic morphology and *ACTB‐GLI1* fusion, managed by thoracoscopic resection and surveillance.

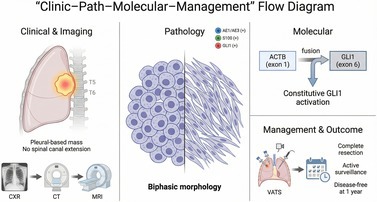

## 1 Introduction

*GLI1*‐altered mesenchymal tumors are a recently recognized group of molecularly defined soft tissue neoplasms characterized by *GLI1* gene fusions or amplification, most commonly involving *ACTB*, *PTCH1*, or *MALAT1* as fusion partners [1, 2, 3]. These tumors exhibit variable morphology, often with biphasic epithelial and mesenchymal differentiation, and are associated with intermediate malignant potential.

While *GLI1*‐altered tumors have been reported across a wide anatomic spectrum—including soft tissue, bone, gastrointestinal tract, and female genital tract—thoracic involvement is rare, and pleural‐based presentations are exceptionally uncommon. To date, only isolated cases involving the lung parenchyma or pleura have been described, most reported as single case reports or small series [4] (Table 1). In this context, additional well‐characterized thoracic cases remain valuable for refining diagnostic criteria and informing management.

**TABLE 1:** Reported thoracic and pleural *GLI1*‐altered mesenchymal tumors.

Author	Site	Fusion	Treatment	Outcome
Kerr et al. [3]	Lung	*PTCH1‐GLI1*	Surgery	Stable
Zhong et al. [4]	Pleura	*ACTB‐GLI1*	Surgery	No recurrence
Present case	Pleura	*ACTB*	Surgery + surveillance	Disease‐free

We describe a pleural‐based *GLI1*‐altered mesenchymal neoplasm harboring an *ACTB‐GLI1* fusion, managed surgically and followed with active surveillance. This case highlights diagnostic pitfalls, molecular correlates, and evolving considerations for targeted therapy in *GLI1* fusion tumors.

## 2 Case Presentation

**FIGURE 1:**
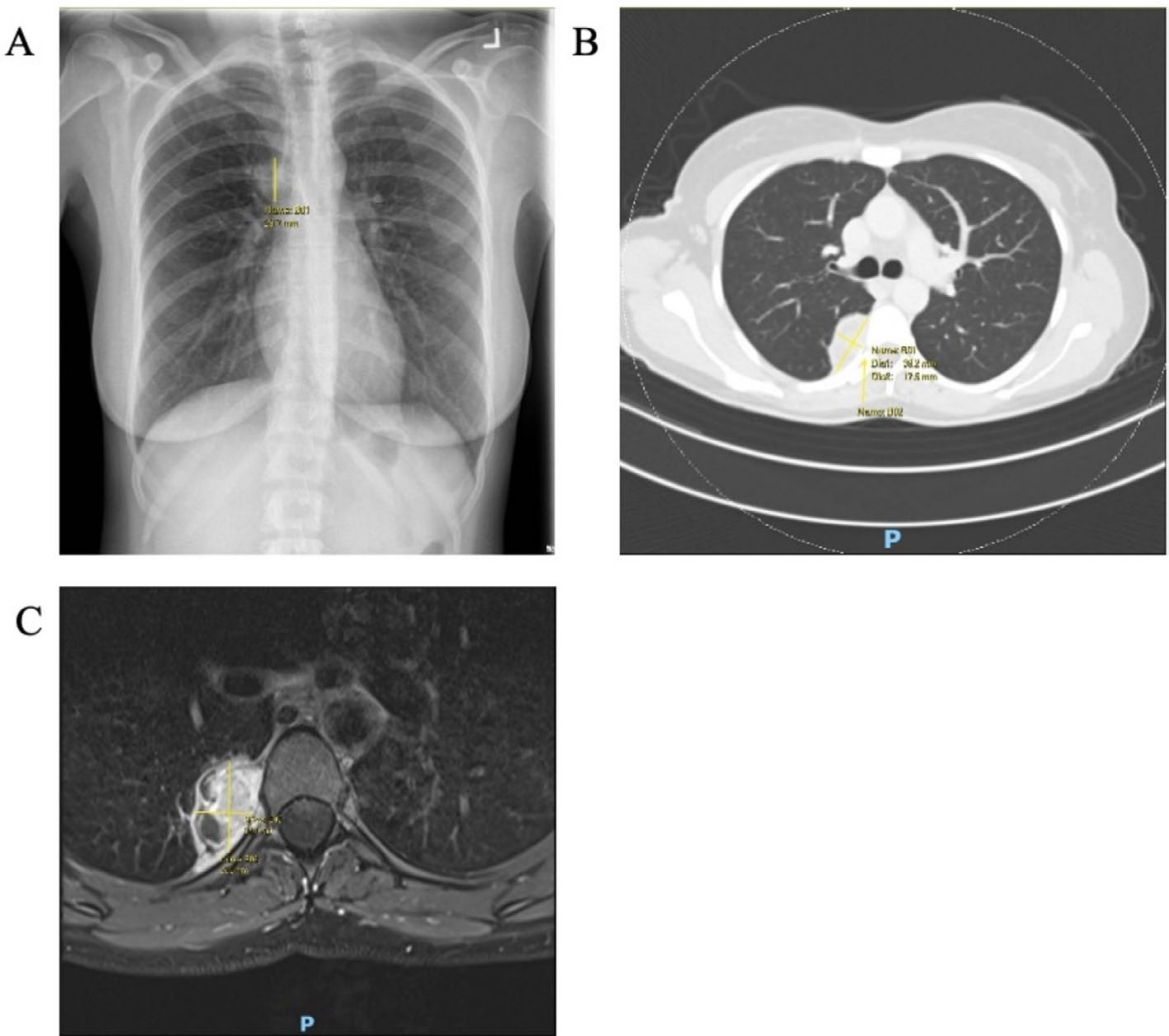
(A) Chest X‐ray showing right upper lobe mass. (B) Contrast‐enhanced CT thorax demonstrating right pleural‐based posterior mediastinal lesion at T5–T6, causing mild rib scalloping without neural foramen extension. (C) Preoperative MRI chest showing multilobulated pleural‐based paraspinal mass at T5–T6 with heterogeneous enhancement and no intraspinal extension.

A 39‐year‐old woman, nonsmoker and previously healthy, was found to have an incidental right upper lobe opacity on routine health screening. Chest radiograph showed a 3.0 cm right upper lobe mass, with otherwise clear lung fields and normal cardiac silhouette, prompting further evaluation (Figure 1A).

Computed tomography (CT) of the chest demonstrated a heterogeneously enhancing right pleural‐based lesion measuring 3.9 × 1.8 cm at the level of T5–T6, along the posterior segment of the right upper lobe and apical segment of the right lower lobe. The mass caused mild compressive atelectasis and scalloping of the right sixth rib near the costovertebral joint, without widening or neural foramen extension. No pleural effusion or mediastinal adenopathy was present (Figure 1B).

Magnetic resonance imaging of the chest confirmed a multilobulated, pleural‐based paraspinal mass with mixed T2‐weighted signal and heterogeneous post‐contrast enhancement, measuring 3.3 × 2.9 × 1.9 cm. There was no intraspinal extension, neural foramen involvement, or vertebral signal abnormality (Figure 1C).

She underwent video‐assisted thoracoscopic (VATS) resection of the mass with wedge resection of the adjacent lung. Intraoperatively, a well‐circumscribed 3 cm lesion was observed close to the sympathetic trunk and the azygos vein and densely adherent to the right lower lobe of the lung.

**FIGURE 2:**
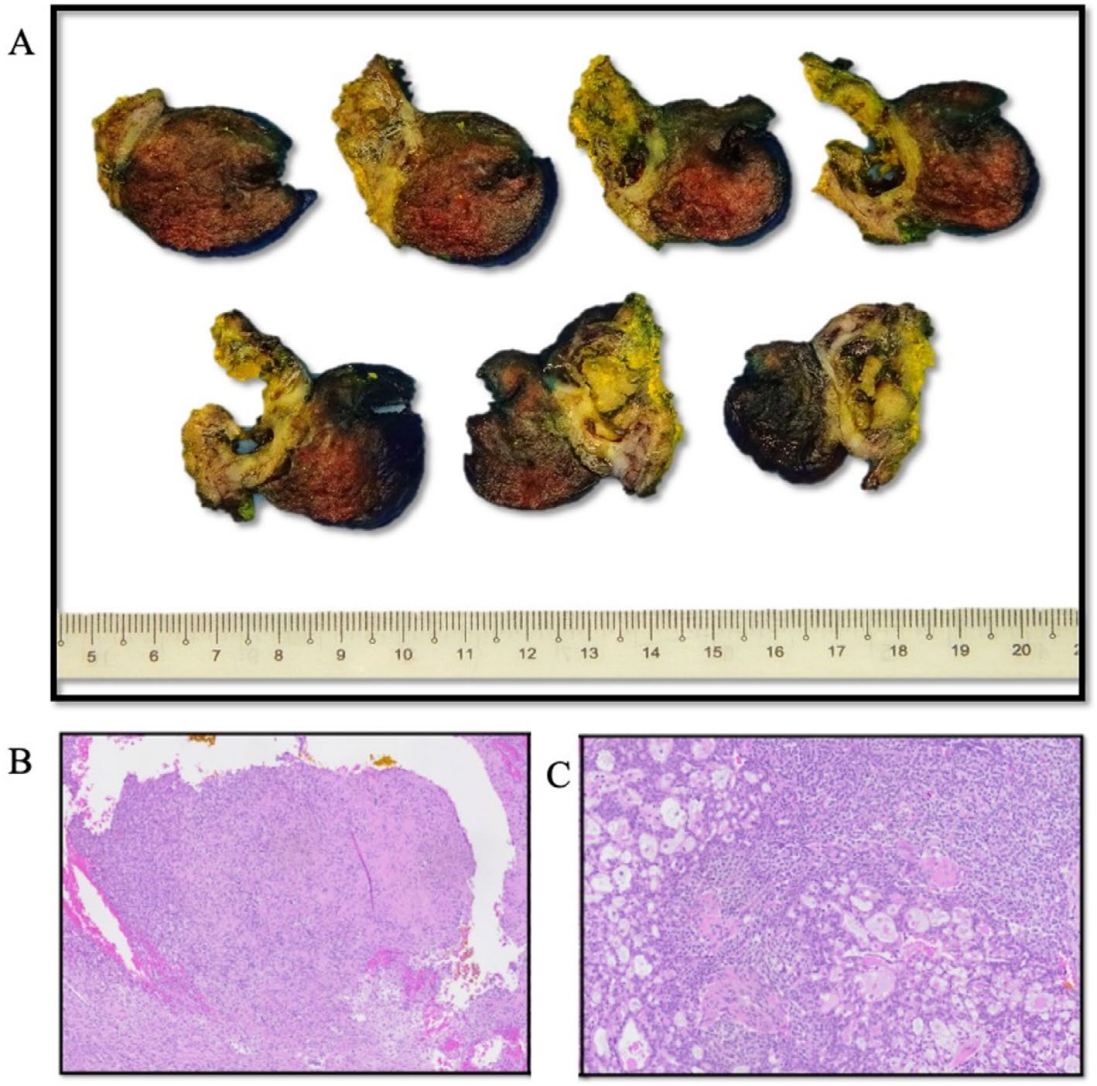
(A) Macroscopic examination revealed a tumor with variable cut surface appearance, ranging from whitish to hemorrhagic, with a pushing margin into the adjacent lung parenchyma. (B) The tumor had a biphasic appearance, with a mesenchymal component ranging from hyalinized areas to spindle cell areas forming short fascicles (H&E ×100 magnification). (C) The epithelioid component comprised nests and trabeculae of relatively monotonous cells. Sieve‐like arrangement with myxoid stromal areas was seen (H&E ×200 magnification).

Macroscopic examination of the mass revealed a tumor measuring 4 × 3.5 × 3 cm. The tumor appeared to have a “pushing” border into the adjacent lung tissue (Figure 2A). The uninvolved lung parenchyma was unremarkable. Microscopically, sections showed a partially encapsulated tumor centered within the soft tissue, with a multinodular growth pattern abutting the pleura. The tumor had a biphasic appearance, with a mesenchymal component ranging from hyalinized areas to spindle cell areas forming short fascicles (Figure 2B). The epithelioid component comprised nests and trabeculae of relatively monotonous cells, featuring round to oval nuclei with a moderate amount of eosinophilic to clear cytoplasm, associated with delicate capillary networks (Figure 2C). The mitotic count was low, approximately 2/10 high power fields. No high‐grade cytologic atypia or necrosis were seen. The tumor abutted the soft tissue margin.

Given the rarity of the entity and diagnostic complexity, GLI1 immunohistochemistry was performed as part of an external expert consultation at Brigham and Women's Hospital, Boston, USA, to support the suspected diagnosis. Immunostaining showed that the tumor cells were strongly positive for GLI1 and S100 proteins, while being negative for SOX10. CD56 was diffusely positive in both epithelioid and spindle cell components. The epithelioid component was positive for pan‐cytokeratin AE1/AE3, as well as CK19 and weak TTF‐1. The Ki67 proliferation index was low, approximately 5%–10% in hot spots. Neuroendocrine markers and myogenic markers, including SMA, were negative. Melanocytic markers such as MelanA and HMB45 were also negative. Representative images from this external immunohistochemical analysis were not available for inclusion.

Given the biphasic morphology and pleural‐based thoracic location, several diagnostic considerations were initially entertained. Synovial sarcoma was considered due to the epithelial and spindle cell components; however, the absence of SS18 rearrangement and lack of diffuse cytokeratin expression in the spindle cell population argued against this diagnosis. Sarcomatoid mesothelioma was excluded based on the absence of calretinin, WT1, and D2‐40 expression, as well as the well‐circumscribed growth pattern without diffuse pleural involvement. Solitary fibrous tumor was considered given the pleural location, but STAT6 immunostaining was negative, and characteristic patternless architecture was absent. Peripheral nerve sheath tumors, including malignant peripheral nerve sheath tumor, were also excluded based on the lack of SOX10 expression and low‐grade histologic features.

**FIGURE 3:**
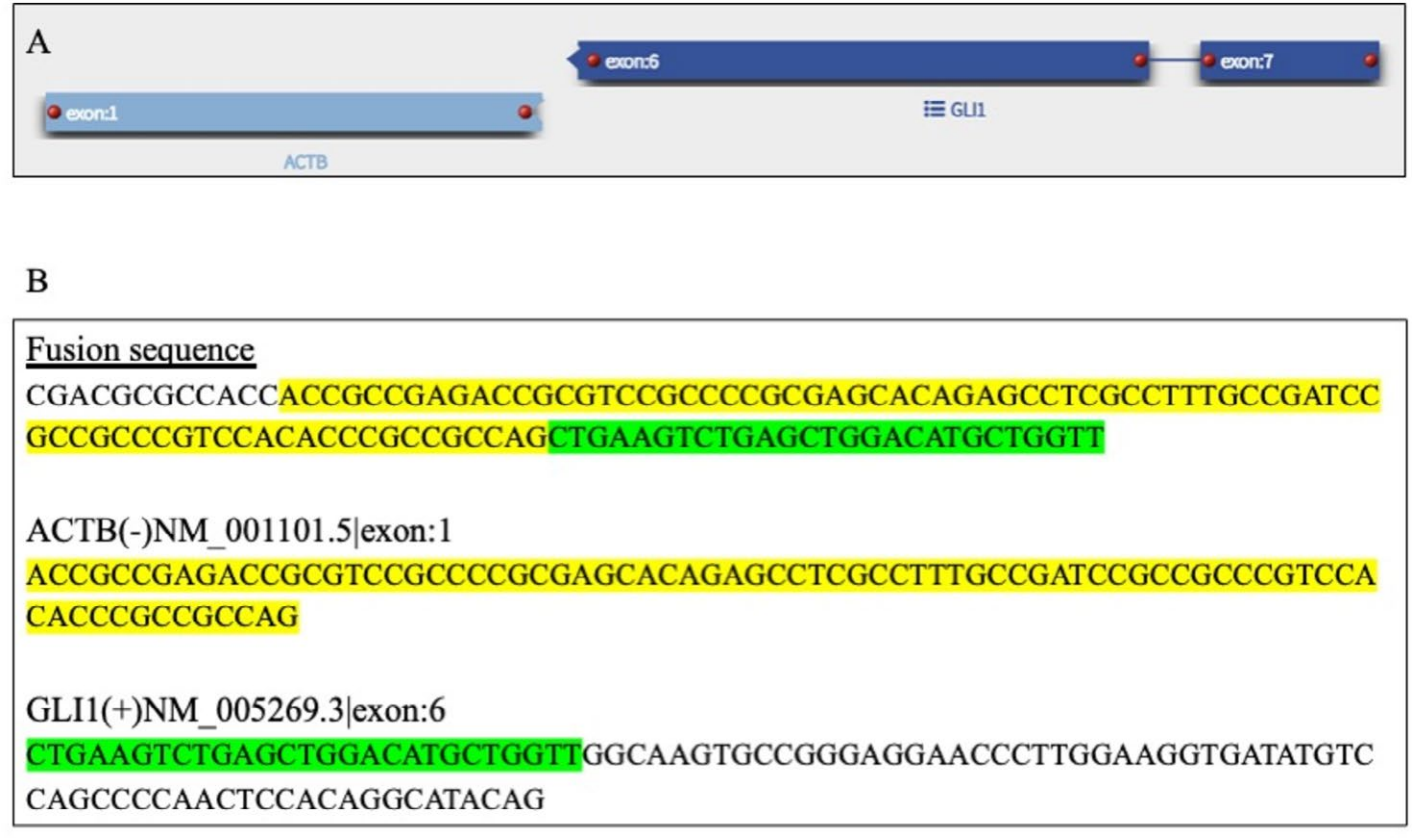
(A) Schematic diagram illustrating the *ACTB* (exon 1):*GLI1* (exon 6) detected using next generation sequencing. (B) Sequences of the fusion gene, exon 1 of the *ACTB* gene, and exon 6 of the *GLI1* gene, respectively.

The combination of biphasic morphology, strong GLI1 immunoreactivity, supportive immunophenotype, and identification of an *ACTB‐GLI1* fusion on next‐generation sequencing established the diagnosis of a *GLI1*‐altered mesenchymal tumor. Next‐generation sequencing using the 129‐gene Archer FUSIONPlex Pan Solid Tumor v2 panel detected an *ACTB* (exon 1):*GLI1* (exon 6) gene fusion (Figure 3), confirming the diagnosis of a *GLI1*‐altered mesenchymal tumor. Expert consultation (Brigham and Women's Hospital) concurred, emphasizing its uncertain malignant potential.

The case was discussed at a multidisciplinary sarcoma tumor board. In view of the close soft tissue margin but absence of metastasis, adjuvant radiotherapy was considered. After detailed counseling, the patient elected for close imaging surveillance. One‐year positron emission tomography/CT demonstrated no recurrence or metastasis. She remains clinically well under ongoing follow‐up.

## 3 Discussion

*GLI1*‐altered mesenchymal tumors represent a recently recognized subset of mesenchymal tumors characterized by aberrant activation of the Hedgehog pathway (HH) [1, 2, 3]. Thoracic involvement remains rare. Reported thoracic cases include tumors arising within lung parenchyma, mediastinum, and, most rarely, the pleural [4]. The *ACTB‐GLI1* fusion leads to constitutive *GLI1* transcriptional upregulation independent of SMO or PTCH1 signaling, driving mesenchymal differentiation [3]. Recent comprehensive reviews have emphasized that these tumors exhibit a broad anatomic distribution, variable epithelial differentiation, and generally indolent to intermediate malignant behavior, although local recurrence and rare metastases have been reported [4, 5].

A recent review of pleural *GLI1*‐altered tumors highlighted their tendency to present as well‐circumscribed masses, often mimicking more common pleural neoplasms radiologically and histologically [4]. The present case aligns with these observations and adds to the small but growing body of thoracic examples, reinforcing the importance of molecular confirmation.

Recently, the biological significance of *GLI1* activation has raised therapeutic interest. Hedgehog pathway inhibitors (HHIs), including vismodegib and sonidegib, are approved for basal cell carcinoma where ligand‐dependent SMO activation predominates [4, 5]. However, GLI1 fusion proteins function downstream of SMO, rendering these agents unlikely to suppress *GLI1*‐driven transcription [6]. Direct *GLI1* inhibitors such as GANT61, Glabrescione B, and related compounds have demonstrated preclinical inhibition of *GLI1*‐DNA binding but remain investigational [7, 8].

Currently, no clinical trial specifically targets the ACTB‐GLI1 fusion protein or other GLI1 fusion proteins [7, 8]. A single case report described prolonged stability of a *PTCH1‐GLI1* fusion‐positive ovarian tumor treated empirically with pazopanib, though this likely reflected nonspecific antiangiogenic effects rather than pathway inhibition [9].

Given the lack of proven systemic therapy, complete surgical excision remains the treatment of choice, and active surveillance is appropriate for localized disease without adverse histologic features. For recurrent or metastatic cases, referral to centers with early‐phase basket trials or molecular tumor boards should be considered. Precise documentation of the *ACTB‐GLI1* fusion variant facilitates eligibility for future studies and collaborative research.

This case adds to the limited number of pleural‐based *GLI1*‐altered tumors and underscores the diagnostic importance of integrating histopathology, immunohistochemistry, and molecular profiling to differentiate them from morphologic mimics such as synovial sarcoma or mesothelioma.

## 4 Conclusion

We report a rare pleural‐based *GLI1*‐altered soft tissue tumor with *ACTB‐GLI1* fusion, successfully treated by thoracoscopic excision and surveillance. Recognition of this entity prevents diagnostic misclassification and overtreatment. Although *GLI1* fusions represent potentially targetable drivers, no validated therapy currently exists, highlighting the need for further translational research and inclusion of such cases in rare tumor registries.

## Author Contributions

M.L. and L.X. wrote the manuscript. C.Y.C. provided histological images and descriptions of pathological findings and revised the manuscript. H.Y.L. provided a molecular description of pathological findings and revised the manuscript. B.‐H.O. supervised the entire manuscript writing. All authors have read and approved the final manuscript.

## Funding

The authors have nothing to report.

## Ethics Statement

The authors have nothing to report.

## Consent

Written informed consent was obtained from the patient to include the information in this manuscript.

## Conflicts of Interest

The authors declare no conflicts of interest.

## Data Availability

The data that support the findings of this study are available from the corresponding author upon reasonable request.
